# Quantifying edge significance on maintaining global connectivity

**DOI:** 10.1038/srep45380

**Published:** 2017-03-28

**Authors:** Yuhua Qian, Yebin Li, Min Zhang, Guoshuai Ma, Furong Lu

**Affiliations:** 1 Key Laboratory of Computational Intelligence and Chinese Information Processing of Ministry of Education, Shanxi University, Taiyuan, 030006, China; 2 School of Computer and Information Technology, Shanxi University, Taiyuan, 030006, China; 3 Research Institute of Applied Biology, Shanxi University, Taiyuan, 030006, China

## Abstract

Global connectivity is a quite important issue for networks. The failures of some key edges may lead to breakdown of the whole system. How to find them will provide a better understanding on system robustness. Based on topological information, we propose an approach named *LE* (link entropy) to quantify the edge significance on maintaining global connectivity. Then we compare the LE with the other six acknowledged indices on the edge significance: the edge betweenness centrality, degree product, bridgeness, diffusion importance, topological overlap and k-path edge centrality. Experimental results show that the LE approach outperforms in quantifying edge significance on maintaining global connectivity.

Many systems in nature and society (such as cellular network, communication network, social network and transportation network) can be described as networks^1^. There is no doubt that the study of complex networks has become a focus. Quite a few measuring methods have been proposed to measure the significance of a node^2^,^3^. However, the study of edges is relatively less, though measuring edge strength has gained attention early.

Researches on edges in complex networks dated from the definition of edge strength proposed by Granovetter^4^ in 1973. The strength of an edge by Granovetter is a linear combination of the amount of time, the emotional intensity, the intimacy and the reciprocal services. Strong edges are usually formed by trusted friends and family, where users tend to be classified into the same cluster. However, weak edges are formed by new acquaintances and people with weak connections.

We intuitively notice that there is a big difference between edge strength and edge significance: strong edges are not always important and weak edges are not necessarily unimportant. Strong edges tend to emerge inside a cluster, while weak edges help to connect two clusters. Experimental and theoretical analysis^5^,^6^ showed that weak edges in the core of a network are attached with high value of significance on maintaining global connectivity. Moreover, in case of weighted networks, the weight of an edge can’t be regarded as edge significance directly. As a result, the lack of reasonable quantization of edge significance will not rise positive effect on generating robust and efficient networks under targeted edge attacks^7^. The “robust yet fragile” property in scale-free networks raised by Albert *et al*.^8^ reveals the phenomenon: scale-free networks display an unexpected degree of robustness, however, these networks are extremely vulnerable to intentional attacks. In realistic networks, a few localized failures or attacks may cause cascading failures and lead to breakdown of the whole system. There has been a lot of research related with analysing infrastructure risk and vulnerabilities base on edge failures^9^,^10^,^11^,^12^. Therefore, it’s more necessary and urgent to carry out the research of edge significance.

Generally speaking, four dimensions, that are edge centrality, weight, tie strength like bridgeness and overlap, diffusion of information, are mainly considered to quantify edge significance. Edge centrality is an indispensable tool in many aspects such as edge significance rank and community detection. In 2002, Girvan and Newman^13^ applied *edge betweenness centrality* to the study of finding and evaluating community structures^14^ in networks. In 2012, Meo *et al*.^15^ developed a *k-path centrality*, initially developed for nodes, which is based on random walks and is defined as the sum of the frequency with which a message traverses an edge *E* from a given source to all k-edges-distance possible destinations. These two centrality measures play a central role in reporting knowledge about data flow in a network. In many unweighted networks, edges are usually represented as binary states, i.e., either present or absent. However, the index of *degree product*^12^,^16^,^17^ offers a quantitative and general approach to understand the complex architecture of real weighted networks. The measuring methods of edge strength can be classified into three categories^18^: topology-based methods^5^,^6^,^19^, attributes-considered methods^20^,^21^,^22^,^23^, behavior sequences focused methods^24^,^25^,^26^. Weak Tie has been tested and proved effectiveness in maintaining global connectivity. The bridgeness^5^,^19^ and topological overlap^6^ are two typical methods based on topology of networks. A bridge, also known as cut-edge, plays an important role in ensuring the network connectivity and different forms are proposed^19^. The index of *bridgeness* developed by Cheng *et al*.^5^ is a classical form, which aims to detect edges between two cliques. The measurement of *topological overlap* by Onnela *et al*.^6^ performs quite well in mobile communication networks. Besides, diffusion of information on edges has undeniable effect on edge significance. Many related works have been carried out to explain the special diffusion effect from the view of edges^27^,^28^. Liu *et al*.^29^ provided the *diffusion importance* of edges in his research on influence maximization^30^ recently. However, the measurements mentioned above are not able to notice the fact that edges with significance on maintaining global connectivity have a critical property based on community structure.

Here we focus on the information of topology in networks, so six acknowledged indices of measuring edge significance are used as benchmarks: *edge betweenness centrality, degree product, bridgeness, diffusion importance, topological overlap* and *k-path edge centrality*. It’s inevitable that each has its own shortcomings. Based on topological information, we propose an approach named link entropy (LE) to quantify the edge significance on maintaining global connectivity. Experiments show that the framework outperforms in measuring the edge significance on maintaining global connectivity.

## Empirical measurements

In this section, we will list six representative structural topology-based indices for undirected and unweighted networks on edge significance. For each index, its shortcomings and its applicable situations are also talked about.

### Edge betweenness centrality

**Figure 1:**
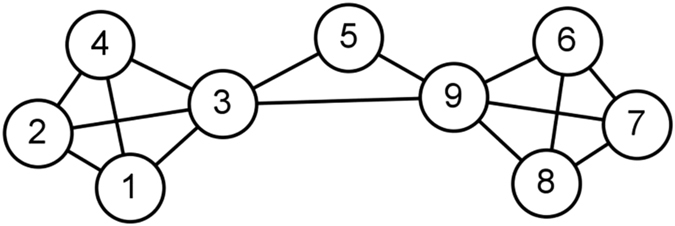
An example for calculating edge betweenness centrality. Although the inter-clique edges *e*_3−9_, *e*_3−5_ and *e*_5−9_ play an important role in maintaining two cliques, the values of edge betweenness centrality of *e*_3−5_ and *e*_5−9_ are lower than that of the inner-clique edges such as *e*_3−4_, which are less important in maintaining global connectivity.

Edge betweenness centrality counts the shortest paths between a pair of nodes passing through the edge, as


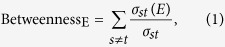


where *σ*_*st*_ is the number of shortest paths from node *s* to node *t*, and *σ*_*st*_(*E*) is the number of shortest paths from *s* to *t* that pass through edge *E*. Notice that it counts the shortest paths between each pair of nodes, so computing complexity is remarkably high. Besides, the value of some edges even key edges may be relatively low (see Fig. 1). What’s more, it not practical to give total burden of information flow to the edge *e*_3−9_ without taking the path *e*_3−5−9_ into consideration.

### Degree product

Degree product is defined as


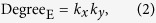


where *k*_*x*_ and *k*_*y*_ are the degree of node *x* and *y* respectively. For an extended form as (*k*_*x*_*k*_*y*_)^*θ*^, *θ* is a tunable parameter. Here we just care about the order of the edges, so the value of *θ* is of no great importance and we just take *θ* as 1 to compute the edge significance. The computation of index only needs the degree of each node, which is quite easy to get. It works well in assortative networks where nodes with high degree tend to connect each other. As to disassortative networks where nodes with high degree tend to connect nodes with low degree, its quantification of edge significance is not so reliable.

### Bridgeness

The edges between two cliques are bridgenesses, where a clique of size *k* is a fully connected subgraph with *k* nodes. It is defined as


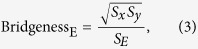


where *x* and *y* are the two endpoints of an edge *E*, and the clique size of a node *x* or an edge *E* is defined as the size of maximum clique that contains this node or this edge. Note that the index only goes for dense networks where similar or relevant nodes are apt to connect and form local clusters such as social and document networks. Furthermore, it is time-consuming to find maximum clique of each node and each edge. In many cases, quite a few edges are measured to be the same value so we can’t distinguish the significance between them.

### Diffusion importance

The diffusion importance of an edge takes disease spread process into consideration. For an edge *e*_*xy*_, when disease spreads along it, there are two possible directions. In one direction, the disease originates from node *x* and spreads along *e*_*xy*_ to node *y*, and then spreads to the other parts of the network through node *y*. So does the spread mechanism in the other direction. In that sense, the diffusion importance of edge *e*_*xy*_ is defined as


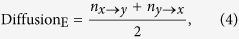


where *n*_*x*→*y*_ is the number of links of node *y* connecting outside the nearest neighborhood of node *x*. The value of index is inevitable to be misled by degree of node somehow: an edge with one high-degree node and one low-degree node may have higher value of edge significance than its real effect when the edge is in the periphery of the network.

### Topological overlap

The topological overlap is a classical measurement based on topological structure. It was initially applied to quantitative study on tie strength in a mobile communication network. Since tie strength is tightly associated with common friends, the topological overlap is defined as


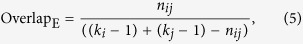


where *n*_*ij*_ is the number of common network neighbours of *i* and *j*, and *k*_*i*_ denotes the degree of node *i*. The index mainly focuses on the probability that the neighbours of two endpoints of an edge are the same. However, there are always the same local structure of an edge whether the edge is in the core of a network or not, which results in the loss of location information.

### k-path edge centrality

Different from the way of propagation in the edge betweenness centrality, information in social networks propagates not only along shortest paths in fact. The k-path centrality index, which is based on the propagation of messages inside a network along paths consisting of at most k edges, simulates message propagations through random walks. It is defined as


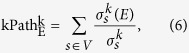


where *s* are all the possible source nodes, *σ*_*s*_^*k*^(*E*) is the number of k-paths originating from *s* and traversing the edge *E* and, finally, *σ*_*s*_^*k*^ is the number of k-paths originating from *s*. For feasible application, ERW-Kpath(Edge Random Walk k-Path Centrality) is adopted to efficiently compute edge centrality values. Since the calculation of ERW-Kpath relies on random walks, it would be fine to fix k as high as possible and better discriminate edges with high centrality from edges with low centrality can be able to obtained. Therefore, in presence of low values of k, edge centrality indexes tend to edge flatten in a small interval and it is harder to distinguish high centrality edges from low centrality ones.

## Link Entropy (LE)

It is undeniable that using more information of social network such as personal information and historical activities can contribute to better quantitative indicator of maintaining global connectivity. In fact, edge attributes like communication time and node attributes are not easy to obtain. Therefore, it’s very important to focus on how to make full use of the topological information and especially how to choose and take advantage of key information.

Notice the fact that an edge between two communities is significant on maintaining connectivity for the components. As to an edge inside the community, there are other paths reachable between two endpoints when it breaks down. So edges between communities are supposed to get more attention. What’s more, how to quantify the significance of an edge is a key point. First we conduct Strategy 1—NMF(Nonnegative Matrix Factorization) according to network topology. Based on the result of the NMF, we apply Strategy 2—QS(Quantification Strategy) to calculate the LE values of all edges to quantify their significance on maintaining global connectivity.

### Strategy 1—NMF(Nonnegative Matrix Factorization)

A network can be modelled as a graph *G* = (*V, E*), where *V* is a set of n nodes and *E* is a set of m edges. In this paper, we assume *G* is an undirected and unweighted graph whose adjacency matrix can be represented as a nonnegative symmetric binary matrix **A**. The element *a*_*ij*_ = 1 if and only if there exists an edge between nodes *i* and *j*, and *a*_*ii*_ = 1 for any 1 ≤ *i* ≤ *n*.

We assume that the pairwise interactions described in **A** are influenced by an unobserved expectation network 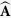, where 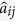 is an observed variable which denotes the probability of existing a connection between nodes *i* and *j*. Here we define *x*_*ik*_ as the probability that node i belongs to community k. So an expected edge 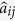 can be estimated as


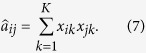


Using the matrix form, the above formula can be rewritten as


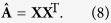


As a result, we can use NMF(Nonnegative Matrix Factorization) method^31^ to get **X**. We make use of square loss function to measure the difference between the observed matrix **A** and the expected matrix 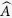, and define the following optimization problem


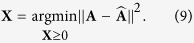


By using gradient descent method introduced by Wang *et al*.^32^. We can obtain the multiplicative update rule for *x*_*ik*_ as


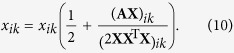


The factorization could be viewed as a representation of the data in a new space of lower dimensionality of k. **X** and **X**^T^ are considered equivalent. So here we can employ any of them to describe the probability distribution of each node in clustering partition, that is, each row denotes the probability of belonging to different community. For a given value of the NMF dimensionality *K*, the algorithm starts with random matrix **X** and each element in **X** is not less than 0. Note that each row in **X** should be normalized to ensure that the sum of each row should be 1 in each iteration. NMF-based algorithms^33^,^34^,^35^,^36^ have gained considerable attention and have been applied to community detection due to its meaningful probability distribution.

### Strategy 2—QS(Quantification Strategy)

Since each row of **X** indicates the probability distribution of the corresponding node, we could find the most uncertain nodes based on the values of **X**. Furthermore, the edges linked with uncertain nodes are usually those between communities, which are significant on maintaining connectivity. To design a quantitative measure to rank the significance of edges, we make use of information entropy and Jensen-Shannon divergence of the node probability distribution. The former aims to find out overlapping nodes, while the latter focuses on measuring the divergence between two probability distributions. The method of Jensen-Shannon divergence is adopted to find the edges between two low-information-entropy nodes, which obviously belong to two different communities. Information entropy and Jensen-Shannon divergence used in this paper are as follows:


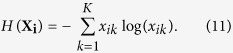



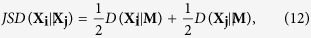


where


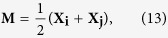


and


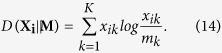


We proposed an approach named LE(link entropy) to quantify the edge significance on maintaining global connectivity. The LE approach takes two situations into consideration. On the one hand, if an edge is linked with an overlapping node, the edge is very likely to be between components, which is of great significance on maintaining connectivity. Based on the information entropy of a node, we just average the values of information entropy of two endpoints to deal with the first situation. On the other hand, when the boundaries of communities is quite clear, that is to say, the information entropy of nodes is low, then the way of handling the first situation doesn’t work. For example, we get **X** by using Strategy 1, where the number of community *K* is set to be 2. Assume the probability distribution of node *a* and *b* is [0, 1] and [1, 0] respectively in extreme circumstance, which means node *a* belongs to the second community and *b* belongs to the first community. Besides, the information entropies of node *a* and *b* are 0. In this case, the way in the first situation become useless when an edge is between them while the edge also locates between two communities. So we need to take the probability distribution between pairwise nodes into consideration. In the paper, Jensen-Shannon divergence is adopted to compensate for the deficiencies of the first situation.

Combined with the above equations (11, 12, 13, 14), we can get it more clearly. The equation (11), which is the formula of calculating information entropy, is used to quantify the degree that a node belongs to different communities. The sum can be obtained as the result of information entropy by running over all communities that a node may belong to. Overlapping nodes would be assigned high values of information entropy. The rest equations (12, 13, 14) aim to deal with the second situation mentioned above. In fact, the Jensen-Shannon divergence in (12) is a variant of Kullback-Leibler divergence like the form in (14). Here we adopt the Jensen-Shannon divergence, subject to symmetry of distance. Hence, the relative entropy of two distributions can keep consistency because their baselines **M**, i.e. equation (13), are the same in (14).

To facilitate understanding, we sort ideas mentioned above as the following three views. Firstly, the edges linked with overlapping nodes are of great significance on maintaining global connectivity. Secondly, the edges between the boundaries of communities are of great significance on maintaining global connectivity. Thirdly, the larger the value of LE is, the greater the edge significance is.

Ultimately, we just average the value that we get in the above-mentioned two aspects as the value of edge significance on maintaining global connectivity for convenience:


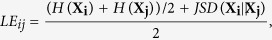


where *H*(**X**_**i**_) is the information entropy of node *i*, **X**_**i**_ is the probability distribution of node *i*.

In experiment we set the parameter *K* to be 2 in Strategy 1. To ensure that the information entropy *H* and the Jensen-Shannon divergence 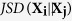 are within interval [0, 1], the base of logarithm is set to be 2 accordingly. Besides, in order to avoid the situation when 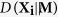 is unable to calculate in case when *m*_*k*_ is zero, we set the zeros in **X** to be 10^−7^, hence 10^−7^ should be taken off from the probability value of the other component in the corresponding row.

### An example of measuring edge significance by the LE

**Figure 2:**
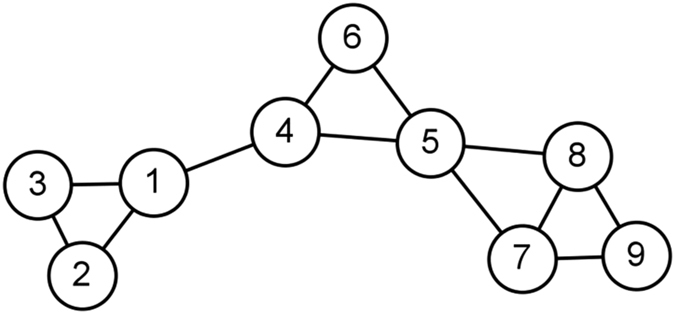
Illustration of calculating the LE.

In Fig. 2, the LE value of each edge is shown in descending order: *LE*_46_ = 0.4994, *LE*_45_ = 0.4778, *LE*_56_ = 0.4259, *LE*_14_ = 0.3744, *LE*_57_ = *LE*_58_ = 0.2139, *LE*_12_ = *LE*_13_ = 0.0147, *LE*_78_ = 5.8143 * 10^−8^, *LE*_89_ = *LE*_79_ = 3.2653 * 10^−8^, *LE*_23_ = 1.0898 * 10^−12^. The larger its LE value is, the greater the significance of the edge is.

### Applying the LE to real collaboration network

Collaboration network is a typical form that most researchers focus on. Different from early collaborative relationship, scholars in different research subjects tend to collaborate with each other, which forms more edges in collaboration network than those of other networks with the same number of nodes. The boundary between real research groups has become increasingly blurred. A node with a high degree is usually a leader in research group or an expert in a particular field. The collaboration between high-degree nodes in different research fields tends to generate creative ideas and influential academic papers. In a sense, the collaboration has great significance on maintaining global connectivity.

Here we crawled collaboration network data with the limit that the number of nodes is not more than ten thousand from the *Microsoft Academic* (https://academic.microsoft.com). Due to the large number of collaborative relations for renowned scholars, one possible case in crawled network is that all scholars may surround the start node. In addition, it’s unnecessary to crawl all relations for each scholar because the low frequency of co-publication may be meaningless. So we crawl top five percent relations for each person by using breadth-first search algorithm. Finally, nodes with degree value of one are removed for the sake of better display effect and less computing load. As a result, the collaboration network is composed of 7147 nodes and 83552 edges.

**Figure 3:**
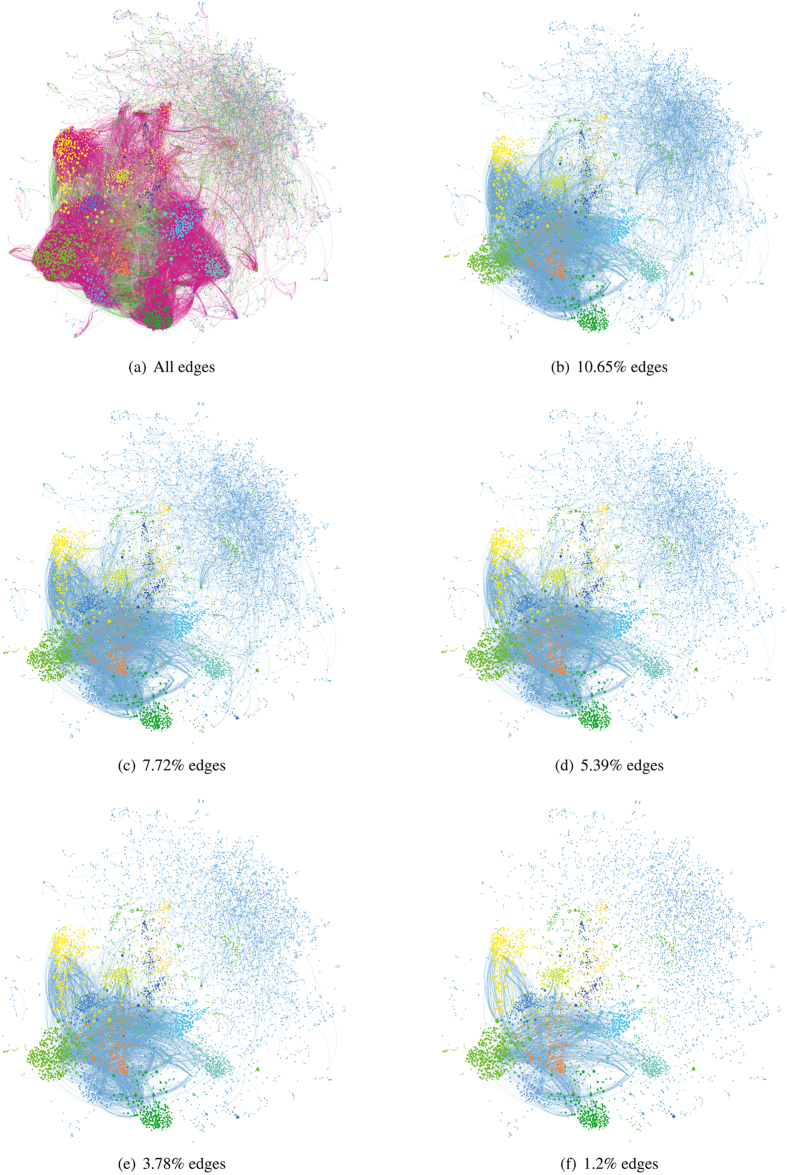
Collaboration networks with different proportions of edges. Subgraphs from (**a**) to (**f**) are the same collaboration networks but with different proportions of edges, where the nodes with the same color are in the same community detected by a heuristic method based on modularity optimization and the node size is proportional to its degree value. In subgraph (**a**), the color of edges varies from red to green along with values measured by the LE in ascending order. Based on (**a**), we obtain subgraphs (**b**–**f**) by removing different percentage of edges in ascending order.

We first calculate the LE values of edges in the real collaboration network by applying the LE method without taking the weight of co-publication into consideration. Then we rank all the edges according to their LE values in descending order. Figure 3(a) shows the distribution of measured LE values for all the edges. Red edges which are assigned smaller values always appear inside the communities, while green edges with larger LE values are inclined to show up in the areas between different communities. In Fig. 3(a), the color of edges varies from red to green along with the increase of values measured by the LE method.

Based on Fig. 3(a), we obtain Fig. 3(b–f) by removing different percentage of edges from the network in ascending order, which show the phase transitions of network when the number of preserved edges decreases gradually. The nodes with the same color are in the same community, which is detected by a heuristic method based on modularity optimization. Besides, the node size is proportional to its degree value.

As shown in Fig. 3(b–f), the edges with relatively large value are the links between high-degree nodes that belong to different communities or fields. What’s more, the number of collaborations between different research fields is quite large in Fig. 3(f). At the same time, we can see from the side, it is a way to enhance influence for scholars.

## Experiments and Discussion

To test the edge significance on maintaining global connectivity, we compared the robustness of each network under seven measurements by successively removing edges in descending order. In the process of removing edges, original networks begin to collapse into pieces, during which some giant components will emerge. In the progress of the edge percolation^37^, two indices are used to capture the critical points. The first one is the fraction of nodes contained in the largest connected component, denoted by *R*_*GC*_. As the fraction of removed edges *f* grows, A sudden decline of *R*_*GC*_ will be observed if the network disintegrates. The other one is the so-called *normalized susceptibility*, defined as


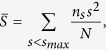


where *N* is the size of the whole network, *n*_*s*_ is the number of components with size *s* and the sum runs over all components but the largest one. Usually, an obvious peak can be observed that corresponds to the precise point at which the network disintegrates. More specifically, the *f* corresponding to the peak is the threshold of edge percolation, i.e., the critical point above which there is no significant giant component. On the whole, if the networks collapse faster, and what’s more, if the peak of 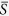 comes earlier and the value of peak is higher, the measurements of edge significance on maintaining global connectivity are more efficient. Here we just calculate indices based on the original network without considering synergistic effects caused by the removing of an edge in the process of each step.

### Data

We conduct experiments on twelve networks for the sake of generality.

- The karate network is the network of friendships between the 34 members of a karate club at a US university.
- The dolphins network is an undirected social network of frequent associations between 62 dolphins in a community living off Doubtful Sound, New Zealand.
- The adjnoun network is the network of common adjective and noun adjacencies for the novel “David Copperfield” by Charles Dickens.
- The celegansneural network is a weighted, directed network representing the neural network of C. Elegans, here we treat it as an unweighted, undirected network.
- The lesmis network is the weighted network of coappearances of characters in Victor Hugo’s novel “Les Miserables”, here we take it as an unweighted network.
- The polbooks network is compiled by Valdis Krebs, where nodes represent books about US politics sold by the online bookseller Amazon.com and edges represent frequent co-purchasing of books by the same buyers.
- The football network is the network of American football games between Division IA colleges during regular season Fall 2000.
- The jazz network is the collaboration network of jazz musicians, where two musicians are connected if they have played in the same band.
- The polblogs network is a political blog network formed by weblogs on US politics and hyperlinks between them.
- The USAir network is a transport network of air lines.
- The Yeast network is formed by protein interactions.
- The power network is an undirected unweighted representation of the topology of the Western States Power Grid of the United States.

### Statistical properties

The basic statistics of these networks is shown in Table 1. *N* and *E* denote the number of nodes and edges in the original network, while 〈*k*〉 and *k*_*max*_ are average degree and maximum degree respectively. The degree heterogeneity is defined as *H*_*k*_ =〈*k*^2^〉/〈*k*〉^2^ that evaluates the heterogeneity of degree sequence of a network. In addition, the degree assortativity *r* is negative, which implies that nodes with large degrees are inclined to connect to nodes with small degrees. On the contrary, the assortativity is positive or close to zero, which implies nodes with large degrees are inclined to connect to each other or connect randomly. The clustering coefficient *C* reflects the degree of node aggregation based on closed triples.

### Comparison between different measurements

**Figure 4:**
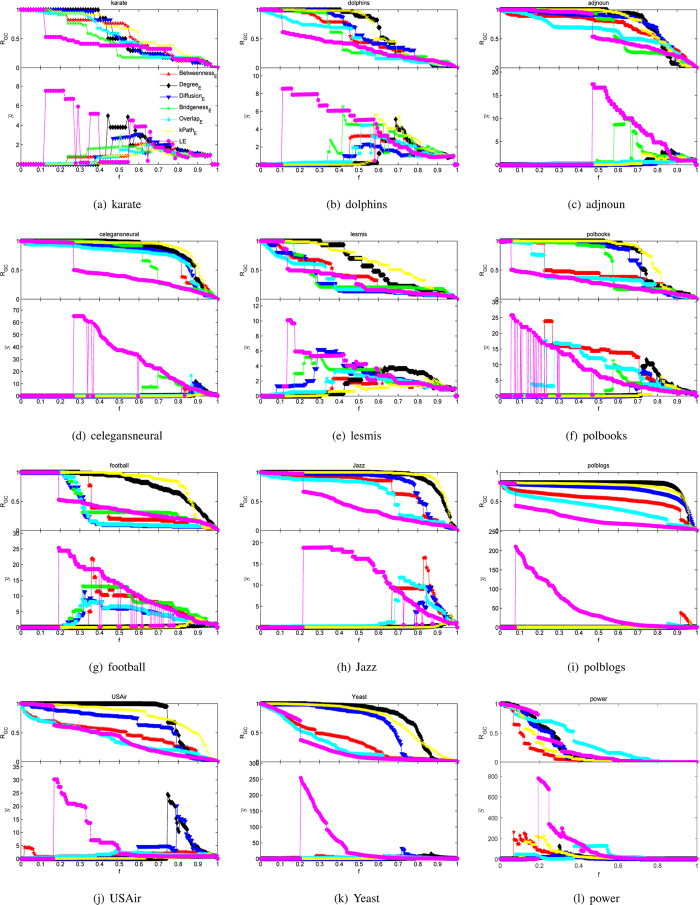
Edge percolation results of different measurements on twelve real networks. From plots (**a**) to (**l**) are the karate, dolphins, adjnoun, celegansneural, lesmis, polbooks, football, Jazz, polblogs, USAir, Yeast, power network, respectively. Seven curves are corresponding to the seven structural indices in plots (**a**–**g**), while the index of bridgeness isn’t shown in the other networks because of its complexity.

The comparison results of twelve networks under different measurements are shown in Fig. 4, among which the index of bridgeness doesn’t appear in some networks because of not being able to get results in tolerable time.

In experiments, here we set *K* = 2 in Strategy 1. Correspondingly, to ensure that the value ranges of information entropy and Jensen-Shannon divergence are between 0 and 1, namely, *H, JSD* ∈ [0, 1], we set base of logarithm to be 2. As for the maximum length of random walks in the *k-path edge centrality*, we choose a value of 20, which is suggested in the original.

As shown in Fig. 4, the upper part of each sub-figure shows the changing process of the largest component *R*_*GC*_ with the rise of removed edges *f*, and the lower part is the curves of *normalized susceptibility* 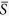 reflecting the process of edge percolation. As we can see, the LE performs almost best in the process of edge percolation except in 4(j) and 4(l) and the peak of the LE is the highest of all. The computing complexity of betweenness is much higher than the LE and the peak of it isn’t as high as that of the LE, though it performs best in 4(j) and 4(l). Above all, one can conclude that the LE is remarkably better than the other six indices in characterizing the edge significance on maintaining the global connectivity.

In detail, the size of the largest connected component remains the same even half of edges are removed, which means nodes can be reachable by other paths. Notice that the worst performer goes to the measurement of degree product in most cases because it’s impossible that edges with significance on maintaining two components are all linked with high-degree nodes. In some cases, there is even no obvious peak in curve of degree product. Compared with the degree product, the indices of diffusion importance and overlap perform much better, which owe much to taking the neighborhood information of the two endpoints into consideration: the former focuses on the number of different neighborhoods, while the latter focuses on the proportion of co-neighborhoods. Furthermore, quantification by global measurement such as the edge betweenness centrality is proved to be more effective although its complexity is much higher. As to the bridgeness, most of the computing time is spent on finding maximum cliques due to rigour definition of the clique. It works well even better than the edge betweenness. The last index, k-path edge centrality, performs unsatisfactorily or as bad as degree product. Maybe the proper length of random walks depends much on a given network. As mentioned above, the LE integrates superiority of NMF and entropy, in which the former strategy orientates edges between components while the latter strategy helps to quantify the edge significance by probability distribution of its two endpoints from two aspects. That’s why LE improves a lot in the process of edge percolation.

### Impact of *K* on the LE

**Figure 5:**
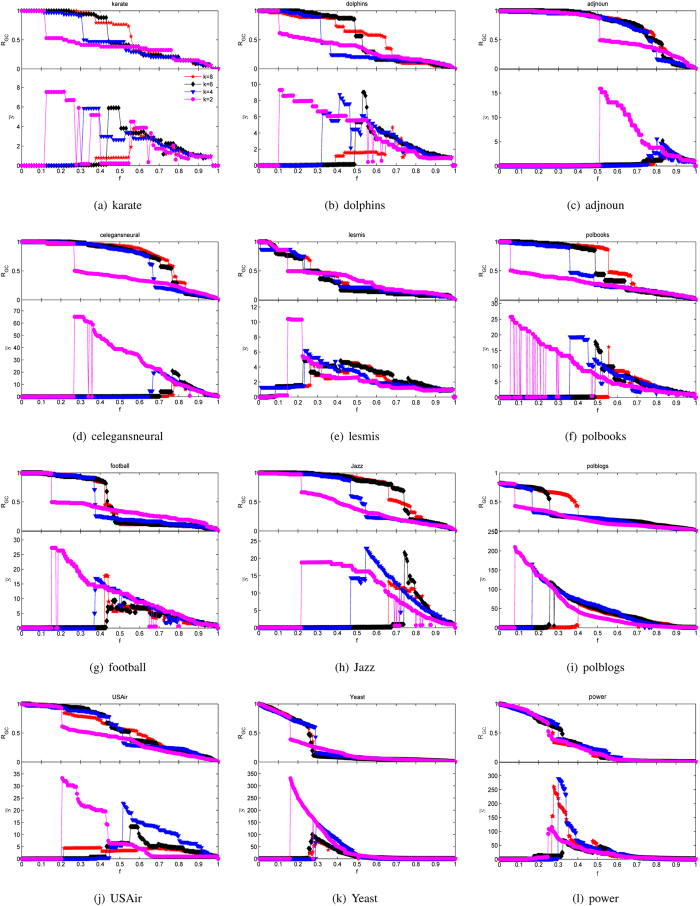
Edge percolation results of different values of *K* in the LE on twelve real networks. Four curves are corresponding to the various *K* ranging from 2 to 8 by step of 2.

How much impact it will have on the LE if *K* changes in NMF? Here *K* ranges from 2 to 8 by step of 2 so that a comprehensive observation can be obtained and the results are shown in Fig. 5. In order to make the LE of different *K* comparable, *H, JSD* should be normalized within [0, 1] for different values of *K*, and the base of logarithm should be set as *K*. We can see no matter how many real communities a network has, the LE performs relatively better when *K* is set to be 2. The cost of removing edges between two communities at that time is less than that of other situations.

In general, there is always more than one community in any realistic networks according to the density of edges. So, the value of *K* = 2 is quite easy to meet. The purpose of our approach is to quantify all edges of the network on the basis of their aptitude of maintaining global connectivity. To verify its effectiveness, we simulate targeted edge attacks by successively removing edges in descending order to get the threshold of edge percolation, at which point the giant component will emerge. In a sense, this problem can be referred to as finding the minimal edge set to disconnect networks as severely as it can be. Hence, the value of *K* = 2 is most likely to achieve the purpose: Firstly, the outcome as two large disconnected components instead of some small fragments is the maximum destruction that can be caused. Secondly, the boundary between communities could be relatively small, i.e., the cost of removing edges is much low. Therefore, the choice of *K* = 2 is valid and feasible.

### Impact of initial value X on the LE

**Figure 6:**
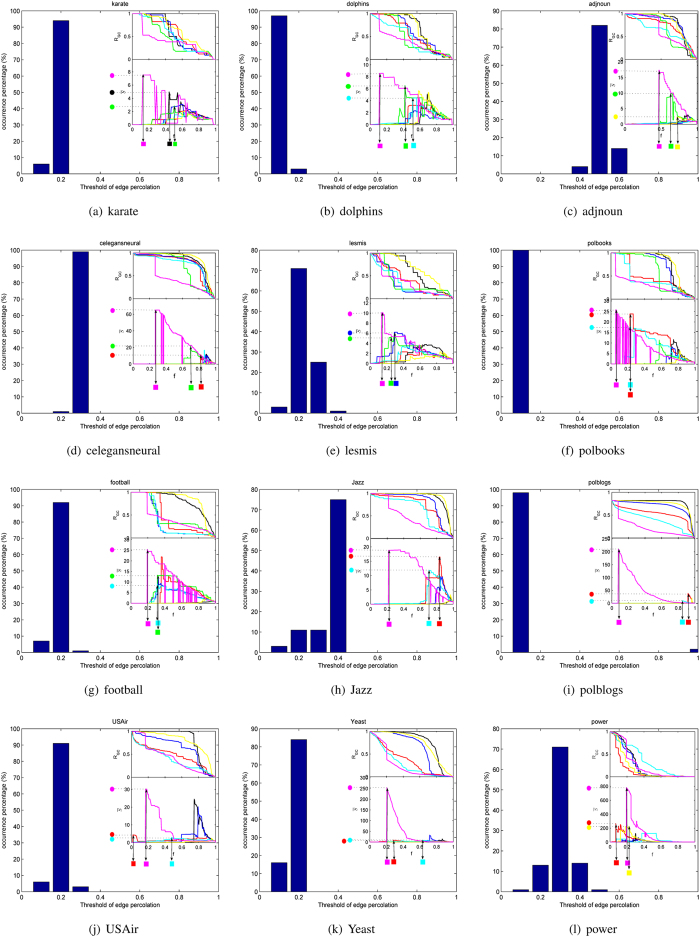
Distribution of thresholds of edge percolation with 100 random initial values of *X* in the LE on twelve real networks. Each value of bar is the percentage of occurrences corresponding to different thresholds of edge percolation of the LE. Since the edge percolation thresholds of the LE in Fig. 4 are just one random occasion compared with Fig. 6, each sub-figure in Fig. 4 is added as a bin to its corresponding sub-figure of Fig. 6. Moreover, for better observation, the locations of edge percolation thresholds and peaks of the top three measurements with relative small thresholds in added bins are marked.

As we all know, the optimal solution in Strategy 1, which is based on NMF, depends much on its initial value **X**. The reason is that the gradient descent method in NMF can just find local best. So we would like to know the impact on the LE if **X** changes. Here we replicate the experiments in twelve networks for 100 times to get the thresholds of edge percolation, and we display the distribution of them in the interval [0, 1] in Fig. 6.

As we can see in Fig. 6, 100 thresholds of edge percolation of the LE for each network is shown in Fig. 6(a–l). In each sub-figure, the horizontal axis is the thresholds of edge percolation that may occur. Each probability in the horizontal axis is a representative with the fluctuation of 0.05. For example, the bar corresponding to the value 0.2 on the horizontal axis represents the number of edge percolation thresholds which are in interval [0.15, 0.25]. The vertical axis represents the occurrence percentage of different thresholds.

Compared with Fig. 6, the edge percolation threshold of the LE in each network in Fig. 4 is just one random occasion. To make comparison between Figs 4 and 6 straight forward, we add each sub-figure in Fig. 4 as a bin to its corresponding sub-figure of Fig. 6. Moreover, for better observation, the locations of edge percolation thresholds and peaks of the top three measurements with relative small thresholds in added bins are marked. The solid rectangles below horizontal axis in each bin have the same color with corresponding measurement, and the black straight line with double arrows connects the peak of curve and the colored rectangle. Correspondingly, the solid circles on the left of vertical axis in each bin have the same color with corresponding measurement, and the dashed line connects the peak of curve and the colored circle. So the top three measurements and their key locations can be well shown.

We can easily find that the edge percolation threshold of the LE in each bin almost falls in the interval with the highest occurrence percentage respectively. Overall, the threshold of edge percolation of the LE is much lower than those of the other methods. In detail, the worst case, i.e., the largest threshold that occurs in Fig. 6, is also much better than the thresholds of edge percolation of the other methods such as in (a), (b), (d), (h) and (k). In Fig. 6(c) and (g), the worst case of the LE is as good as the other measurements at least. Notice that the largest thresholds in Fig. 6(i) and (e) perform worst especially in 6(i). However, the percentage of the worst cases in Fig. 6(i) and (e) is no more than three percent, whose effect can be ignored. As to Fig. 6(j) and (l), the lowest threshold of the LE is still larger than the threshold of edge betweenness, which implies that the index of edge betweenness is suitable to networks with no obvious communities. However, the peak of the LE is much higher than that of edge betweenness with small increasement of *f* in bins of (j) and (l). In a word, the LE approach is more versatile and performs better in networks with obvious communities where the establishment of an edge depends on the similarity of nodes. Besides, random initial value of **X** is feasible for the LE to quantify the edge significance on maintaining global connectivity.

## Conclusion

Analyzing and profiling the structures of real networks is an important step in understanding and controlling dynamic behaviors on networks. Inspired by the established systems of measuring the significance of nodes and the strength of edges, we believe the edge significance on maintaining the global connectivity is an important research area. In this article, we proposed an approach called LE(link entropy). Compared with six acknowledged topological indices, firstly, the speed of disintegration in the edge percolation process by the LE is much faster, that is to say, the size of largest connected component is much smaller after removing the same number of edges, secondly, the threshold of edge percolation is much lower and the peak corresponding to its threshold is the highest, indicating that the LE performs best in characterizing the edge significance. Meanwhile, we discuss the impact of *K* and initial value **X** in the LE. Empirical analysis reveals it’s better to set *K* as 2, which means the cost of removing edges between two communities is less than other situations. In summary, the index of LE is more effective to help us in some real-life applications such as controlling the spreading of diseases or rumor and withstanding the targeted edge attacks especially in networks with obvious communities. Moreover, the application of LE can also be generalized to other network configurations such as bipartite networks directly.

In addition, we have a try on weighted or directed networks by using the LE directly and the findings are as follows: Firstly, the LE performs not bad, but not as well as that in undirected and unweighted networks. Secondly, the LE still works better than the edge betweenness centrality in networks with obvious communities, but much worse in networks with no obvious communities. After all, the constructing mechanism in Strategy 1 is originally designed for undirected and unweighted networks.

It is worth noticing that the LE is based on the NMF in Strategy 1, which makes it very hard to be applied in the large-scale networks. In order to settle the problem, we may have to profile the hierarchical structures of networks by community detection algorithms at first, and then assign the LE to the detected communities by taking each community as a node, and distribute its value to the internal nodes in each community finally. In essence, the quantification of edge significance has a huge space to improve itself. Moreover, research on more large-scale networks will also be a part of our future work.

## Additional Information

**How to cite this article:** Qian, Y. *et al*. Quantifying edge significance on maintaining global connectivity. *Sci. Rep.* **7**, 45380; doi: 10.1038/srep45380 (2017).

**Publisher's note:** Springer Nature remains neutral with regard to jurisdictional claims in published maps and institutional affiliations.

## Figures and Tables

**Table 1:** Basic statistics of the twelve networks.

Networks	*N*	*E*	〈*k*〉	*k*_*max*_	*H*_*k*_	*r*	*C*
*karate*	34	78	4.5882	17	1.6933	−0.4756	0.5706
*dolphins*	62	159	5.1290	12	1.3268	−0.0436	0.2590
*adjnoun*	112	425	7.5893	49	1.8149	−0.1293	0.1728
*celegansneural*	297	2148	14.4646	134	1.8008	−0.1632	0.2924
*lesmis*	77	254	6.5974	36	1.8273	−0.1652	0.5731
*polbooks*	105	441	8.4000	25	1.4207	−0.1279	0.4875
*football*	115	613	10.6609	12	1.0069	0.1624	0.4032
*Jazz*	198	2742	27.6970	100	1.3951	0.0202	0.6175
*polblogs*	1490	16715	22.4362	351	3.6218	−0.2212	0.2627
*USAir*	332	2126	12.8072	139	3.4639	−0.2079	0.6252
*Yeast*	2375	11693	9.8467	118	3.4756	0.4539	0.3057
*power*	4941	6594	2.6691	19	1.4504	0.0035	0.0801

Structural properties include number of nodes (*N*), number of edges (*E*), average degree (〈*k*〉), maximum degree (*k*_*max*_), degree heterogeneity (*H*_*k*_), degree assortativity (*r*), clustering coefficient (*C*).
